# Unexpected Headless and Tailless Fish in the Stomach Content of Shortfin Mako *Isurus oxyrinchus*

**DOI:** 10.1371/journal.pone.0088488

**Published:** 2014-02-12

**Authors:** Sebastián Biton Porsmoguer, Daniela Bănaru, Philippe Béarez, Ivan Dekeyser, Manuel Merchán Fornelino, Charles F. Boudouresque

**Affiliations:** 1 Aix-Marseille University, UM 110, MIO (Mediterranean Institute of Oceanography), Campus de Luminy, Case 901, Marseille, France; 2 Asociación CHELONIA, C/Aristóteles, 3, Madrid, Spain; 3 Muséum National d'Histoire Naturelle, UMR 7209, Département Ecologie et Gestion de la biodiversité, Paris, France; Aristotle University of Thessaloniki, Greece

## Abstract

The stomach content of 113 individuals of shortfin mako *Isurus oxyrinchus* was analyzed. Individuals were sampled at landing in Vigo (Spain) and captured by sea-surface long-liners in the vicinity of the Azores Archipelago and between Azores and the Iberian Peninsula, in March and October 2012, and March 2013. Teleosts constituted the dominant item, mainly Atlantic saury *Scomberesox saurus* (87% of teleost prey). Among them, 94% were deprived of both head and the caudal fin, while the flesh and bones of the body were preserved. The presence of eye's lenses, the number of which was consistent with the number of fish remains, likely rules out the elimination of the heads before ingestion. There is no obvious explanation for this unexpected and unrecorded pattern of digestion.

## Introduction

Long-line Spanish and Portuguese fleets which exploit offshore northeastern Atlantic waters (15°–35°W and 30°–45°N) target swordfish *Xiphias gladius*, tuna *Thunnus obesus, T. albacares, T. alalunga* and *Katsuwonus pelamis* and the Atlantic bonito *Sarda sarda*, species of high market value. Two pelagic sharks, blue shark *Prionace glauca* and shortfin mako *Isurus oxyrinchus*, theoretically constitute accessory catches. However, their mass exceeds that of the target species. For the past 5 years, 2008–2012, they represent 64 and 10%, respectively, of the fishery landings at Vigo, Spain. Swordfish, tuna and Atlantic bonito only constitute 23.2 and 1%, respectively, of the landings (Xunta da Galicia, i.e. regional government, pers. comm.).

The shortfin mako is one of the most common predatory elasmobranchs of the world ocean. It dwells in tropical and temperate areas, in the epipelagic and mesopelagic zones, down to 150 m depth [1].

Sharks, and in particular *Isurus oxyrinchus*, are slow-growing, long-lived, low fecundity and late sexual maturity species, which characterizes K strategists [2]. They are therefore highly vulnerable species. The steady increase in shark fishery has led to overexploitation of their stock in several areas. This is currently the focus of considerable international concern [3].

Knowledge of the diet of a species provides a basis for understanding its interactions with other species and its role in the foodweb of the ecosystem [4]. The diet of the shortfin mako has been investigated by a number of authors, e.g., in the northwestern Atlantic [5], the northeastern Atlantic [6]; [7], southeastern Atlantic [8], southwestern Atlantic [9] and the Pacific Ocean [10]; [11]; [12].

The above-mentioned studies show that teleosts and cephalopods are the dominant prey in the shark diet. The goal of the present work was to evidence the possible peculiarities of the diet of juvenile individuals in the northeastern Atlantic Ocean. In this area, though of major importance for Spanish and Portuguese fishery, their diet has been poorly studied. In the framework of this study, we observed an unexpected process of fish digestion, beginning by the heads and tails, so that nearly all teleost individuals were headless and tailless and studying vertebrae was the only way to identify them.

## Materials and Methods

### Ethics Statement

The shortfin mako *Isurus oxyrinchus* is globally assessed as a vulnerable species, according to the IUCN Red List criteria, on the basis of estimated and inferred declines [13]. However, it is not listed in the Annexes of the European Union (EU) Habitats Directive [14].

At the 17^th^ meeting of the Contracting Parties to the Barcelona Convention, in February 2012, the shortfin mako, together with 9 other shark species, was moved from Annex III (species whose exploitation is regulated) to Annex II (endangered and threatened species).

The Barcelona Convention only concerns the Mediterranean Sea, not the Atlantic. In the Berne Convention for the conservation of European wildlife and natural habitats, which concerns both the Mediterranean and Atlantic European countries, the shortfin mako was placed in Appendix III (protected fauna and flora).

This Appendix does not involve actual protection, in contrast with Appendix II (strictly protected species). Finally, the shortfin mako is not listed in Appendices I, II or III of the Convention on international trade in endangered species of wild fauna and flora [15].

Overall, despite the worldwide dramatic decline of the shortfin mako, especially in the Mediterranean Sea, where it is considered as critically endangered by the IUCN [13], with an over 99.9% loss since the early 20^th^ century [16], the species is still legally fished and marketed in Spain, as in other EU countries.

**Figure 1:**
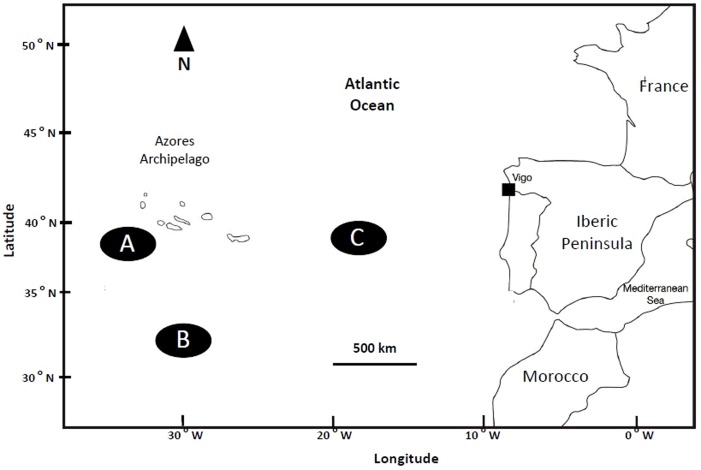
Distribution of the sampling areas (northeastern Atlantic). Zone A (southwestern of the Azores Archipelago), zone B (south of the Azores) and zone C (between the Azores and the Iberian Peninsula).

Catches were made by the Vigo (Galicia, Spain) long-line fleet, in three areas of the northeastern Atlantic (Fig. 1), south-west of the Azores Archipelago (A), south of the Azores Archipelago (B), and between the Azores Archipelago and the Iberian Peninsula (C), in March and October 2012, and in March 2013. The geographical coordinates for locations sampled correspond to the fishing area (Table 1). Sampling was authorized during a meeting in Vigo (Spain), with Mr Carlos Botana (Environment Department) and Mr Luis Pérez (Fishing Department) of the Vigo Harbor Authorities, on 26 January 2012.

**Table 1:** Geographical coordinates of sampled individuals of shortfin mako sharks.

Number of sampled sharks	Geographical Coordinates	Zone
1–35	35–38°05 N – 32–35°25 W	A
36–77	38°00 N – 18°00 W	C
78–113	30–34° N – 30°00 W	B

All shortfin mako were caught with a hook by the fishermen. The cause of death was asphixia. Sharks were refrigerated (∼0°C) and landed from ca. 4 days to several weeks later. Sampling of stomachs was done on dead and refrigerated individuals landed at the Vigo harbor facilities (harbor fish market). The sharks were not killed for the purposes of this study. They are all sold for human food in Spanish and European markets. Samples of stomachs were donated by the Vigo long-line fleet for this study. We did not use a method involving sacrifice of the shortfin mako *Isurus oxyrinchus*.

In Table 2, we present further details regarding shortfin mako feeding [17], [18]. We used certain indices its diet as percentage by number (%N), percentage by weight based on digested non-reconstituted prey (%W) percentage frequency of occurrence (%O) and index of relative importance (%IRI) [19].

**Table 2:** Feeding strategies of *I. oxyrinchus* (n = 113).

*Isurus oxyrinchus* prey groups	%N	%W	%O	%IRI
**Cephalopoda**	14.1	2.4	40.8	19.4
**Cetacea**	2.3	21.6	8.5	1.6
**Chelonia**	0.4	30.5	1.4	0.3
*Caretta caretta*	0.4	30.5	1.4	0.3
**Crustacea**	0.8	0	2.8	0
Decapoda	0.8	0.0	2.8	0.0
**Teleost**	82.4	45.5	77.5	78.7
*Scomberesox saurus*	72.1	9.0	43.6	33.3
Other teleost species	10.3	36.5	33.9	45.4

%N = percentage of number; % W = percentage of not reconstituted prey weight; %O = percentage frequency of occurrence; %IRI = percentage index of relative importance.

### Sampling and analysis

Overall, 113 shark individuals were sampled, originating in the three study areas (Table 3). Total length (TL), from the tip of the snout to the end of the caudal fin, and total mass were measured, before dissection and stomach extraction. Stomachs were then frozen (−20°C) and transported to the MIO (Mediterranean Institute of Oceanography) in Marseille (France). There, stomachs were defrosted, immediately dissected and their content analyzed.

**Table 3:** Number of studied individuals of shortfin mako sharks (*Isurus oxyrinchus*), non-empty stomachs, stomachs with teleosts, percentage of occurrence of teleosts, number of teleost individuals and of *Scomberesox saurus* and number of headless teleost individuals.

Month	Zone	Number of studied sharks	Number of non-empty stoma-chs (ne)	Number of stomachs with teleosts (t)	Percenta-ge of occurrence of teleosts (t/ne ×100)	Number of teleost individu-als (*S. saurus*)	Number of head-less teleost individuals (*S. saurus*)
March 2012	A	35	18	12	66	27 (*17*)	25 (*17*)
March 2013	B	37	24	16	67	39 (*26*)	34 (*26*)
October 2012	C	41	29	27	93	150 (*145*)	148 (*144*)
TOTAL		113	71	55	77.5	216 (*188*)	207 (*187*)

**Figure 2:**
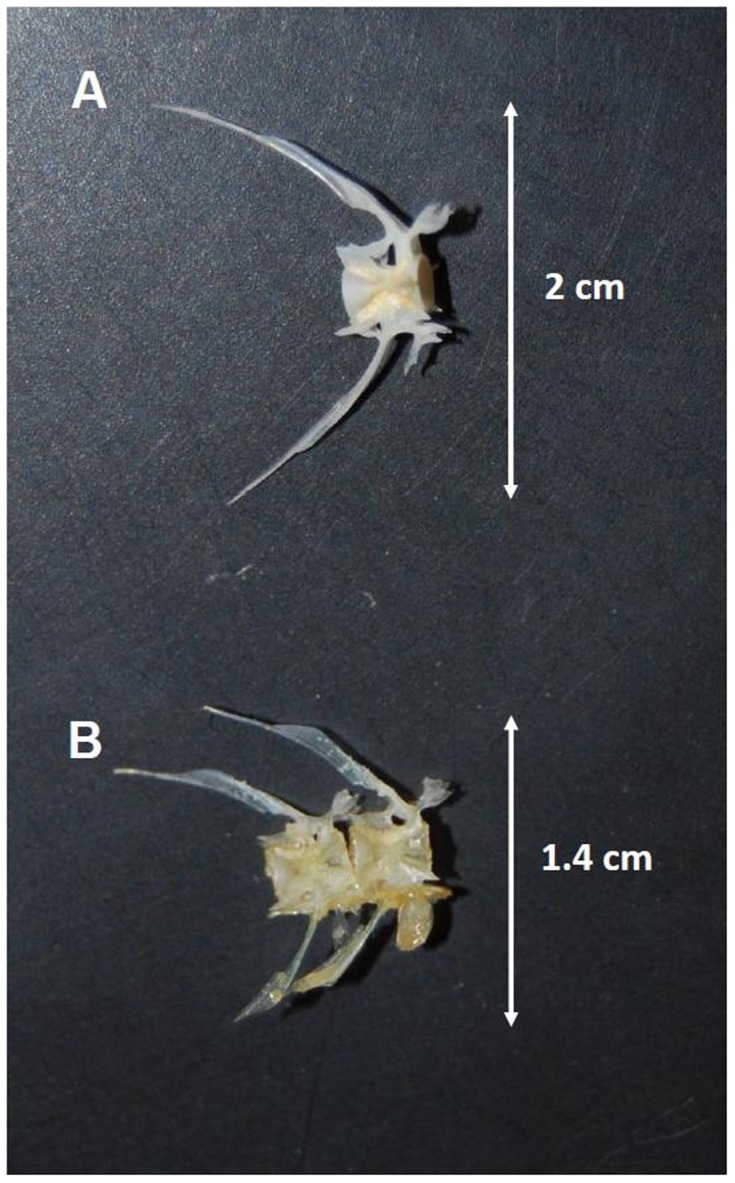
Comparison between a vertebra of a specimen of *Scomberesox saurus*, (MNHN 5227, 341 mm TL) (A) and two vertebrae from a prey of shortfin mako (individual M1, caught at zone A) (B). Photo Sebastián Bitón.

Prey other than teleosts were determined to species or lowest possible taxonomic level, according to their state of digestion. Teleleosts were identified by means of vertebra examination. Vertebrae were compared with the fish osteological collections of the Muséum National d'Histoire Naturelle (MNHN, Paris, France) (Fig. 2). The reason for this identification procedure was that heads were nearly always absent, except in one *Scomberesox saurus* (Beloniformes, Scomberesocidae) and some other teleost specimens.

Even in these cases, macroscopic identification could not be performed due to the advanced state of digestion. In addition, the otoliths were in too poor a condition to be exploited.

## Results and Discussion

Most studied individuals (108 out of 113) were juveniles. TL ranged from 50 to 187 cm. In the shortfin mako, sexual maturity is reached at 195 cm in males and 280 cm in females [1]. A very few adult individuals, whose TL was over these thresholds, were also studied: 205 to 254 cm (4 males, zones B and C) and 340 cm (1 female, zone A). These 5 individuals were included within the considered database, since their stomach contents were consistent with those of the majority of the juvenile individuals in the area.

Among the 113 stomachs studied, 42 were empty, the vacuity ratio being therefore 37.2%. Overall, 216 individuals of teleosts were counted within the 55 stomachs containing teleosts. If sampling zones are considered separately, 27 teleosts were counted in 12 out of 18 non-empty stomachs at zone A (percentage of occurrence: 66%); 39 teleosts were counted in 16 out of 24 non-empty stomachs at zone B (percentage of occurrence: 67%); 150 teleosts were counted in 27 out of 29 non-empty stomachs at zone C (percentage of occurrence: 93%) (Table 3).

We can say that teleost is a main prey consumed by *I. oxyrinchus* (82.4% of total number of prey). Shortfin mako prefers the Atlantic saurus, *Scomberesox saurus*, representing 87% of teleost prey and 72.1% of all prey (Table 2). These single species was present in 63%, 67% and 97% of non-empty stomachs sampled in March 2012 (zone A), in March 2013, (zone B).and in October 2012 (zone C) respectively.

The remaining 13% of teleost prey was constituted by *Balistes capriscus*, *Scomber scombrus*, *Thunnus alalunga, Alepisaurus ferox*, unidentified Bramidae and other unidentified teleost.

The analysis of the shape of the vertebrae allowed identification of this species as the Atlantic saury, *Scomberesox saurus*. This species dwells in shoals near the sea surface, in temperate and tropical waters. It consumes pelagic crustaceans, small teleosts (Clupeidae) and *Noctiluca* (dinoflagellate) [20]. Its trophic level is estimated at 3.6 [21].

**Figure 3:**
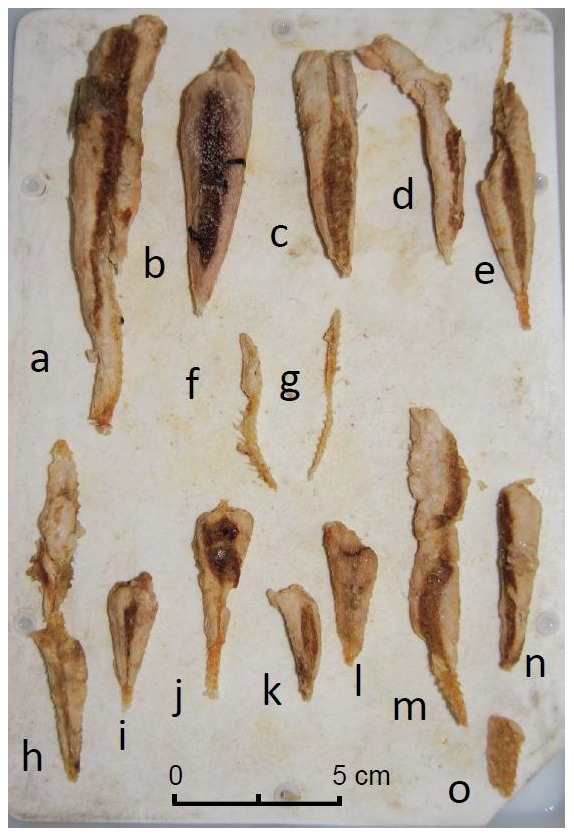
Stomach content of shortfin mako (individual M1, caught at zone A). At least 13 individuals of *Scomberesox saurus* are present (a through e and h through o); f and g, though not counted, might actually represent 2 highly digested individuals (see text). Photo Sebastián Bitón.

Within stomachs, *S. saurus* nearly always occurred with the same appearance, totally deprived of head and tail (caudal fin) (Fig. 3). Only one head (without eyes) and one caudal fin, in an advanced state of digestion, were found (both in a single individual).

The general shape of the fish and the vertebrae made it possible to definitely orient (front vs rear) the fish remains. The head was absent up to, at least, the operculum (e.g. Fig. 3a). Together with the head, the front portion of the ‘body’ (here, ‘body’ means the part between the operculum and the caudal peduncle) was more or less absent (e.g. Fig. 3i, 3j and 3k). The caudal fin was always absent (with the one above-mentioned exception), up to the caudal peduncle. In fact, the part of the fish body which was present was the portion between the caudal peduncle and at least the rear of the body, sometimes part of the body of varying length, very rarely the total ‘body’. The length of the preserved portion of the ‘body’ ranged from 2 to 19 cm. In addition to the body remains, a number of isolated fish crystalline lenses were observed. A total of 542 lenses were collected, which is consistent with the minimum number of individuals (MNI) identified, namely 216.

The fact that the number of lens pairs (270) was slightly over the MNI suggests that the total fish count was only slightly underestimated and therefore more or less correct. Consumed individuals were not fragmented into several pieces, which would have resulted in overestimates, not underestimates; rather, small body debris, not taken into account (Fig. 3f and 3g) corresponds to almost completely digested individuals.

However, it cannot be excluded that the rough correspondence between number of bodies and eyes lenses could be due to different speed in the digestion process of bodies and head, but also differences between different species.

The comparison between the caudal vertebrae of the fish remains with the reference specimen of the MNHN leads us to roughly estimate that the maximum length and mass of the consumed individuals were ∼35 cm and ∼85 g. The maximum length of *S. saurus* in the wild is 50 cm [20].

Remains of the other teleost prey had the head: two individuals of *Balistes capriscus*, two individuals of *Thunnus alalunga*, two individuals of *Scomber scombrus*, one individuals of *Alepisaurus fero*x and one individual of unidentified teleost.

A number of criteria were proposed to characterize the digestion state of prey in stomachs of elasmobranchs [22]. 1: Prey was recently ingested, easy to identify and is all in one piece or bitten in half. 2: Prey is intact or bitten in half and it is possible to take most of the standard measurements. 3: Most of the prey is present, although in various pieces and only one or two measurements can be taken. 4: Measurements cannot be made, some meat pieces still together, loose scales and skeleton pieces united. 5: Random loose pieces (e.g. otoliths, vertebrae, eyes, telson, beaks). 6. Empty stomach or unidentifiable mush. The teleost remains observed here in the studied shortfin mako sharks correspond to state 4 (Fig. 3).

The sequence of digestion and gastric evacuation of foodstuffs in elasmobranchs has not been fully elucidated. Small more friable and easily digestible items are evacuated more quickly than the largest items with lower surface to volume ratios [23]. According to [24], bones are a relatively resistant food fraction contributing to an extended evacuation time. The digestion process encompasses a phase during which a large portion of the food is digested rapidly, then a ‘residual phase’ in which less digestible parts are softened and passed slowly [24], [25]. In the sandbar shark *Carcharhinus plumbeus* feeding on menhaden, *Brevoortia tyrannus*, the time required to completely evacuate 98% of a meal is ∼4 days; this time is consistent with other studies dealing with sharks fed teleosts, e.g. [26]. As far as our results are concerned, the digestion of the bony head before the flesh of the body is surprising. What could be the reason behind this process?

We may explain the phenomenon through three hypotheses.

i. First hypothesis: sharks do not eat the head but only the posterior part of the body, with the tail. In fact, we only found one head decomposed among 188 individuals of *Scomberesox saurus* identified. The Atlantic saury is a species of teleost that lives in schools. It could be difficult for sharks to eat a moving prey entirely. They may bite only the posterior part. The aspect of teleost prey found in stomachs might confirm this theory.

The occurrence of fish eye lenses may either infirm the above mentioned hypothesis since the number of lens pairs roughly corresponds to the number of prey, or confirm it and be due to remains of previous prey already digested. The lenses found may belong to other species consumed before. Concerning the state of digestion of Atlantic saury, we did not find scales and skin, but only part of body with vertebrae protected by muscles (Figure 3). We can confirm with certainty that the tail was decomposed before the rest of body. We found the posterior part of the body of many individuals without tails. However it was impossible to eat this part of the body without the tail. The gastric acid probably digests the tail first.

ii. Second hypothesis: sharks eat the head of the prey. The head together with the tail are not protected by muscles in comparison with the rest of body. Acid gastric juice can decompose it rapidly. On the basis of this theory, we would not find the head but only fish eye lenses (see above). The muscles slow down the digestion process of the bone structure because these were not directly in contact with acid gastric juice.

Protein is the main chemical component of eye lenses and especially γM7-Crystallin [28].We can explain the slow decomposition of lenses because they are composed of a crystalline matrix and an albuminoid element that withstands acid digestion [29].

For teleost prey, we can say that otoliths were digested before the eye lenses. Some works on the variation in gastric acid secretion in sharks show that continuous acid secretion may increase digestive efficiency [30]. We could not measure the gastric pH in stomach but some works concerning similar pelagic species [30], showed that the gastric pH can increase to 8.2–8.7 in the 2–3 days after feeding. Gastric pH can have periodic oscillations that range from 1.1 to 8.7 when the stomach is empty [30].

iii. Third hypothesis: the absence of head and tail could be an artifact due to a *post-mortem* particular digestion process occurring between capture and landing. The last hypothesis was ruled out by the occurrence of mackerel (*Scomber scombrus*) with preserved head and tail not digested in stomachs, corresponding to the bait used by fishermen. We can therefore consider that the digestion process stops with death.

Overall, the two first hypotheses or a combination of both are the most probable.

## Conclusion

To our knowledge, the occurrence of teleosts deprived of both the head and the tail, while the body (bone and flesh) was more or less preserved, in the stomach of the shortfin mako *Isurus oxyrinchus*, as well as in other predatory sharks, has not previously been reported. Most authors do not or only poorly describe the state of digestion of the teleost prey, with the exception of isolated fish lenses (e.g. [27] for *Prionace glauca*; [11] and [6] for *Isurus oxyrinchus*). We cannot speculate upon the general occurrence of this process for the studied species, the shortfin mako and the main observed prey: *Scomberesox saurus*, or for the other 5 prey species. However, it is worth noting that the phenomenon of headless teleosts was observed in all but 9 stomachs containing teleosts, at the two study seasons and the three study zones.
